# Completion of the genome sequence of Oidiodendron maius splipalmivirus 1

**DOI:** 10.1007/s00705-024-06126-z

**Published:** 2024-09-16

**Authors:** Stefania Daghino, Marco Forgia, Massimo Turina

**Affiliations:** 1 Institute for Sustainable Plant Protection, CNR, Strada delle Cacce 73, 10135 Torino, Italy; 2 Department of Plant Protection, School of Agriculture, The University of Jordan, Amman, Jordan

## Abstract

Mycoviruses with an unprecedented genome organization, featuring the RNA-directed RNA polymerase (RdRp) palm domain coding sequence being split into two distinct genome segments, have been found recently in a few fungi and oomycetes of different lineages and have been proposed to be named “splipalmiviruses”. One of these, Oidiodendron maius splipalmivirus 1 (OmSPV1), has been detected in the ericoid mycorrhizal fungus *Oidiodendron maius*, and it has been proposed to be bisegmented. Here, we complete the genome sequence of this virus by describing a third RNA segment, which is 2000 nt long and whose terminal sequences are identical to those of the other two segments of OmSPV1. This segment contains a single open reading frame that codes for a protein with unknown function and has a low level of sequence identity (47%) to the putative protein encoded by the third segment of another splipalmivirus from *Magnaporthe oryzae*: Magnaporthe oryzae narnavirus virus 1 (MoNV1). Based on these features, we propose the RNA segment to be the third segment of the OmSPV1 genome.

Mycoviruses are currently classified by the ICTV into 23 families including a total of 206 species [1], the majority of which have either a double-stranded (ds) or single-stranded (ss, positive or negative) RNA genome, while only three ssDNA mycoviruses have been characterized so far [2]. Mycoviruses have been found in recent years in all lineages within the kingdom Fungi and the class Oomycota in the kingdom Chromista, including organisms characterized by different trophic strategies, such as phytopathogens, mycorrhizal fungi, endophytes, entomopathogens, human pathogens and saprophytes, including edible fungi [3–5].

Around 80% of vascular plants can establish mycorrhizal symbiosis with fungi [6], and while a few of these fungi have been found to host mycoviruses, only in some cases does the virus modulate the host phenotype [7]. The first report of mycoviruses from ericoid endomycorrhizal fungi [8] indicated the presence of an ourmia-like virus and a narna-like virus, tentatively named "Oidiodendron maius splipalmivirus 1" (OmSPV1) because of its genomic organization, in which the region encoding the RNA-directed RNA polymerase (RdRp) palm domain is split into two genomic segments (OmSPV1-RNA1 and OmSPV1-RNA2).

Mycoviruses with this unusual genomic organization in which the RdRp is encoded on two distinct segments have been found in fungi and oomycetes belonging to different trophic guilds (plant pathogens, saprotrophs, and mycorrhizae), and they can have two [8, 9], three [10], four [10–15], or more [10] genome segments. Based on whether the RdRp core motif B is present on the RNA1 or RNA2 segment, their replicases have been classified as type I or type II divided RdRps [13]. The currently known type I RdRps, including the one from OmSPV1, form a monophyletic clade in the phylum *Lenarviricota* that is closer to the *Narnaviridae* than to the *Mitoviridae* or *Botourmiaviridae* [10] and consists of three major branches corresponding to three newly proposed genera [14].

OmSPV1 has been detected in three strains deposited at the Mycotheca Universitatis Taurinensis (University of Torino, Italy) under the accession numbers MUT1357, MUT1358, and MUT5500. These strains were isolated from roots of *Calluna vulgaris* collected from different patches in an area close to San Francesco al Campo (Piemonte, Northern Italy [16]). A thorough re-analysis of the metatranscriptomic data from the same work, looking for contigs with 5’- and 3’-terminal sequences similar to those of OmSPV1-RNA1 and OmSPV1-RNA2 revealed the presence of a contig 1981 nt long. A schematic diagram of the proposed complete genome of OmSPV1 is shown in Fig. 1a. The aim of the present study was to complete the genome sequence of OmSPV1 from the fungal isolate MUT1357 with a description of the third genomic segment, hereafter called "OmSPV1-RNA3", including sequencing of the terminal regions, the verification of possible endogenization, and a comparative analysis of the predicted coding sequence with the most similar known predicted proteins from other splipalmiviruses.

**Fig. 1:**
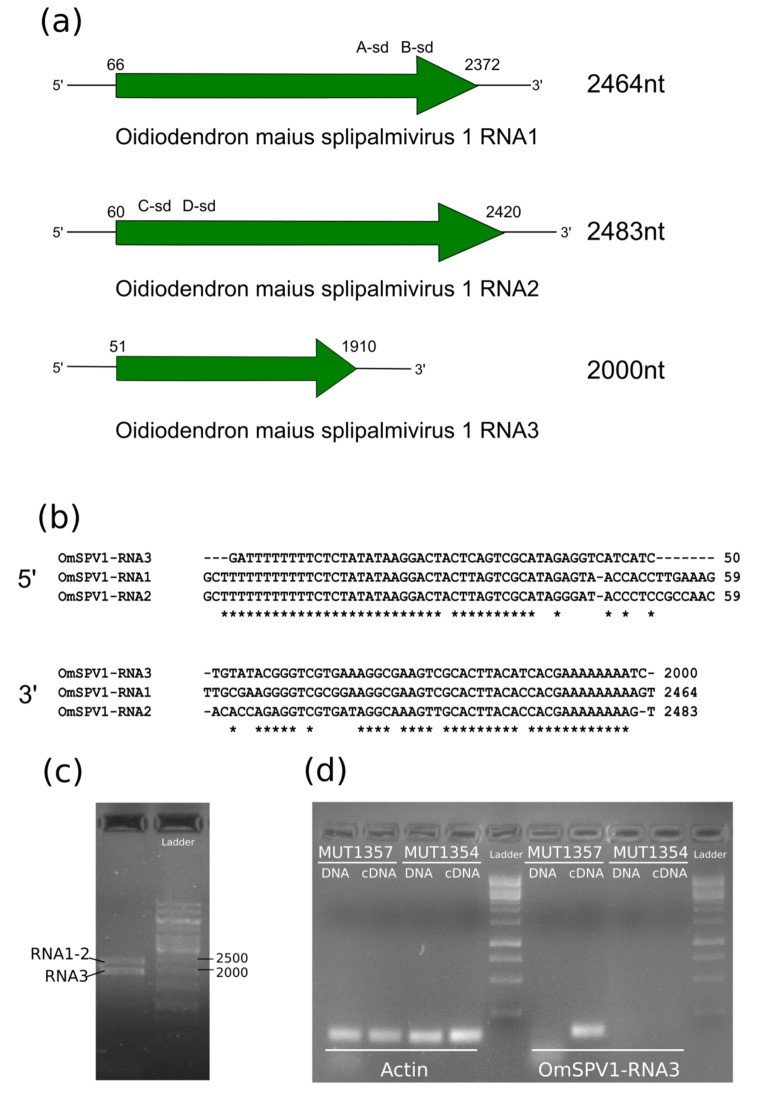
(**a**) Schematic representation of the trisegmented genome of OmSPV1, with the two first segments described previously by Sutela and colleagues [8] and confirmed here by full Sanger sequencing in isolate MUT1357 and the third segment described here. The letters “A-B-C-D” indicate the subdomains (sd) of the putative RdRp palm domain; (**b**) Aligned sequences of the cDNA ends of the three segments of OmSPV1 obtained by RACE. (**c**) PCR amplification of the three OmSPV1 segments (ladder: GeneRuler 1 kb DNA Ladder, Thermo Scientific). (**d**) Gel electrophoresis of the amplicons obtained by qPCR and RT-qPCR, respectively, from DNA and cDNA of the infected strain MUT1357 and the uninfected strain MUT1354, showing the absence of a DNA template for OmSPV1-RNA3. The actin gene was used as a positive control for the genomic DNA (gDNA) amplification from the same extracts (ladder: GeneRuler 1 kb DNA Ladder, Thermo Scientific)

The methods used for RNA extraction, library preparation, sequencing, next-generation sequencing (NGS) data analysis and in vitro validation were reported previously by Sutela and colleagues [8]. The terminal sequences of OmSPV1-RNA3, shown in Fig. 1b, were completed by RACE analysis, using the protocol described by Sutela and colleagues [8] with specific internal primers (for the 5’ end, RNA3_OmSPV1_345Rev [5’-AACGAAGGCACGGATGC-3’] and RNA3_OmSPV1_260Rev [5’-GCTGATCAGCTAGAAAGACGC-3’], and for the 3’ end, RNA3_OmSPV1_1738For [5’-ACGCATCACTGTGTGGTTG-3’] and RNA3_OmSPV1_1704For [5’-CAGGTGGGTGAACTTTCACC-3’]). The conserved nucleotides shared by the three OmSPV1 segments extend downstream of the 5’-poly(U) stretch (29 nt with a polymorphism at the 19th nucleotide) and upstream of the poly(A) stretch (Fig. 1b), suggesting that the three genomic segments are from the same virus. Indeed, the full-length sequence of the three segments could be amplified by RT-PCR using primers designed based on the OmSPV1-RNA1 and OmSPV1-RNA2 ends (Fig. 1c). The three genome segments of OmSPV1 were cloned using a Zero Blunt TOPO PCR Cloning Kit (Thermo Fisher) and sequenced by the Sanger method, confirming the sequence previously predicted in silico and completed in vitro by RACE. The three complete segment sequences have been deposited in the GenBank database with the accession numbers MN736964, MN736965, and MW988098.

The presence in the fungus of a DNA template for the OmSPV1 segment RNA3 (originating from endogenization) was excluded by performing a qPCR assay using the total nucleic acids extracted from strain MUT1357 and comparing the result with the one obtained using cDNA synthesized by RT-qPCR. The *O. maius* strain MUT1354, in which no viruses have been detected [8], was used here as negative control for the reaction. The results showed that the OmSPV1-RNA3 sequence could be amplified only from cDNA (Fig. 1d), like the sequences of OmSPV1-RNA1 and OmSPV1-RNA2 [8].

The genome segments of the proposed splipalmiviruses that do not encode the RdRp, i.e. RNA3 and RNA4, can be either mono- or polycistronic [10, 14]. OmSPV1-RNA3 is predicted to be monocistronic, coding for a single protein with unknown function that showed 47% sequence identity to the protein encoded by the third monocistronic segment of Magnaporthe oryzae narnavirus 1 (MoNV1, with 98% query coverage according to a BLASTp search of the nr database of viral sequences; Fig. 2a). The tertiary structure of the two proteins was predicted by Alphafold network [17] and aligned by using PyMOL (The PyMOL Molecular Graphics System, Version 1.2r3pre, Schrödinger, LLC). This analysis showed a poor structural alignment over the full sequence (RMSD = 8.251 Å), but high structural conservation (RMSD = 0.977 Å) was observed over a domain of 85 residues (Fig. 2b and the corresponding red square in Fig. 2a). An identity matrix of the non-RdRp proteins from OmSPV1, MoNV1, and other splipalmiviruses, in which the longer predicted coding ORF of the RNA3 was included, suggests that the OmSPV1 and MoNV1 proteins do not show significant sequence similarity to the others (Fig. 2c). Phylogenetic analyses performed on RNA1 and RNA2 both suggested that OmSPV1 and MoNV1 belong to the same proposed clade, tentatively named “Triasplipalmivirus” [14]. Similarly, other splipalmiviruses assigned to the same tentative clade based on an alignment of their RNA1- and RNA2-encoded proteins [14] also showed the highest similarity of the RNA3-encoded proteins with each other, with a few exceptions, such as SsNV1 and AfNV1. The detection of the third segment of OmSPV1 provides a new complete splipalmivirus genome sequence that will help to further expand and clarify the phylogeny of this group of viruses. It remains to be determined whether the non-RdRp coding segments are essential, and their possible function still needs to be investigated. In addition, the complete sequencing and description of the third segment of OmSPV1 will potentially permit the development of reverse genetic tools that will help to shed light on the functional biology of OmSPV1 and, indirectly, of this new and so far little-understood group of viruses, although our initial attempts to reproduce infectivity through cDNA clones have failed.

**Fig. 2:**
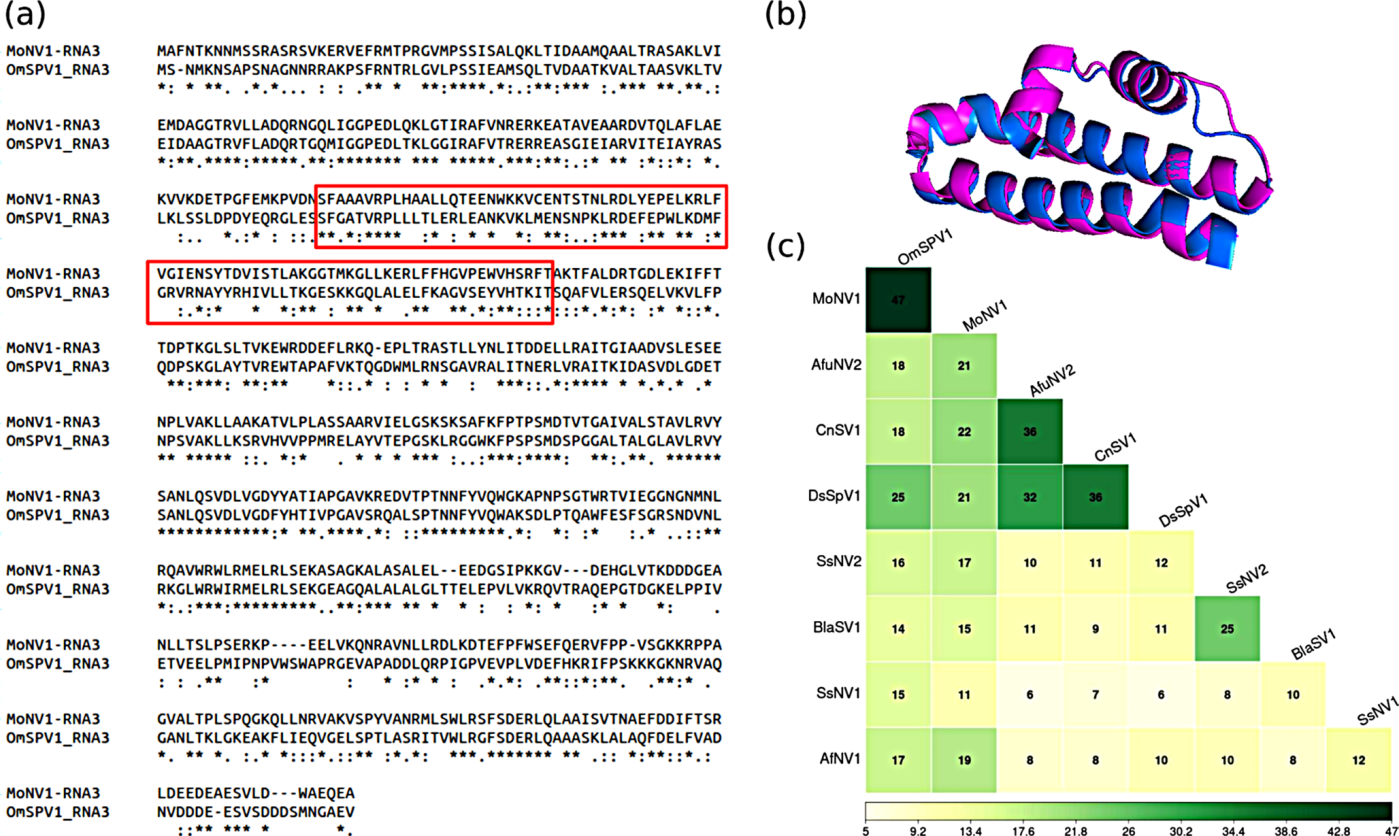
(**a**) Alignment of the ORFs of OmSPV1-RNA3 and MoNV1-RNA3, produced using MAFFT version 7 with default parameters [18]. The red box indicates the best-conserved stretch of amino acids according to an alignment of tertiary structures. (**b**) Alignment of the tertiary structure of 85 residues from OmSPV1-RNA3 (magenta) and MoNV1-RNA3 (blue). (**c**) Percent identity matrix of splipalmiviruses RNA3-encoded proteins, created after sequence alignment by MAFFT v.7 with default parameters [18]. The accession numbers of the proteins included in the identity matrix are as follows: OmSPV1 (Oidiodendron maius splipalmivirus 1), UJT31791.1; MoNV1 (Magnaporthe oryzae narnavirus 1), BCH36657.1; AfuNV2 (Aspergillus fumigatus narnavirus 2), BCH36624.1; CnSV1 (Cryphonectria naterciae splipalmivirus 1), BCX55511.1; SsNV2 (Sclerotinia sclerotiorum narnavirus 2), QZE12028.1; SsNV1 (Sclerotinia sclerotiorum narnavirus 1), QZE12032.1; BlaSV1 (Bremia lactucae associated splipalmivirus 1), OR060921.1; DsSpV1 (Diplodia seriata splipalmivirus 1), UOK20177.1; AfNV1 (Aspergillus flavus narnavirus 1), UAW09580.1

We conclude that OmSPV1-RNA3 is the third genome segment of the putative trisegmented virus OmSPV1. Both in silico searches and amplification by RT-PCR using common terminal sequences and primers failed to reveal the presence of other RNA segments associated with this virus, which can sometimes be associated with other splipalmiviruses [10, 13–15].

## Data Availability

The full OmSPV1 genome sequence is available under the accession numbers MN736964, MN736965, and MW988098.
